# Feasibility of a teacher-supervised ChatGPT-assisted workflow for individualized exercise planning in university physical education: a two-phase study using a fuzzy Delphi process and a cluster pilot trial

**DOI:** 10.3389/fspor.2026.1896856

**Published:** 2026-09-25

**Authors:** Ruiqing Dong, Ying Liang, Jianbo Sun, Fang Chen, Qingzhen Hou, Xiaoqi Wang, Yuanyan Huang, Wenjing Zhang

**Affiliations:** 1 Yunnan Technology and Business College, Kunming, Yunnan, China; 2 Physical Education Institute, Guangzhou College of Commerce, GuangZhou, GuangDong, China; 3 Department of Health Management Center, The Third Affiliated Hospital of Southern Medical University, GuangZhou, GuangDong, China

**Keywords:** artificial intelligence, ChatGPT, exercise prescription, feasibility study, individualized instruction, physical education, pilot trial, university students

## Abstract

**Background:**

University physical education can promote physical activity and fitness, but individualized instruction is difficult to implement in routine classes. This study developed a structured input framework for ChatGPT-assisted individualized fitness teaching and evaluated its feasibility, safety, acceptability, and preliminary effects.

**Methods:**

This two-phase study involved non-sports-major university students. Phase I used a two-round fuzzy Delphi process to develop the framework. Phase II was a 6-week cluster pilot trial involving two intact classes allocated by coin toss to either AI-assisted instruction (*n* = 30) or conventional instruction (*n* = 30). The AI group received teacher-supervised, AI-generated fitness plans. Primary outcomes included feasibility, safety, implementation, and acceptability.

**Results:**

The final framework comprised 49 items, with 96.4% achieving a consensus rate ≥75%. Follow-up completion rates were 96.7% and 93.3% in the AI and control groups, respectively. Attendance, completion rate, and perceived exertion were similar between groups. Time in the target heart-rate zone was higher in the AI group (33.95 ± 4.33 vs. 32.60 ± 3.74 min, *P* = 0.033), as was perceived personalization (4.0 ± 0.7 vs. 3.2 ± 1.0, *P* = 0.001). No serious adverse events occurred, and most physical and functional outcomes did not differ significantly between groups.

**Conclusions:**

ChatGPT-assisted individualized fitness instruction was feasible, safe, and acceptable within a structured teacher-supervised workflow, with its primary short-term benefit being improved perceived personalization.

**Clinical Trial Registration:** Chinese Clinical Trial Registry ChiCTR2500107730.

## Graphical Abstract

**Figure:**
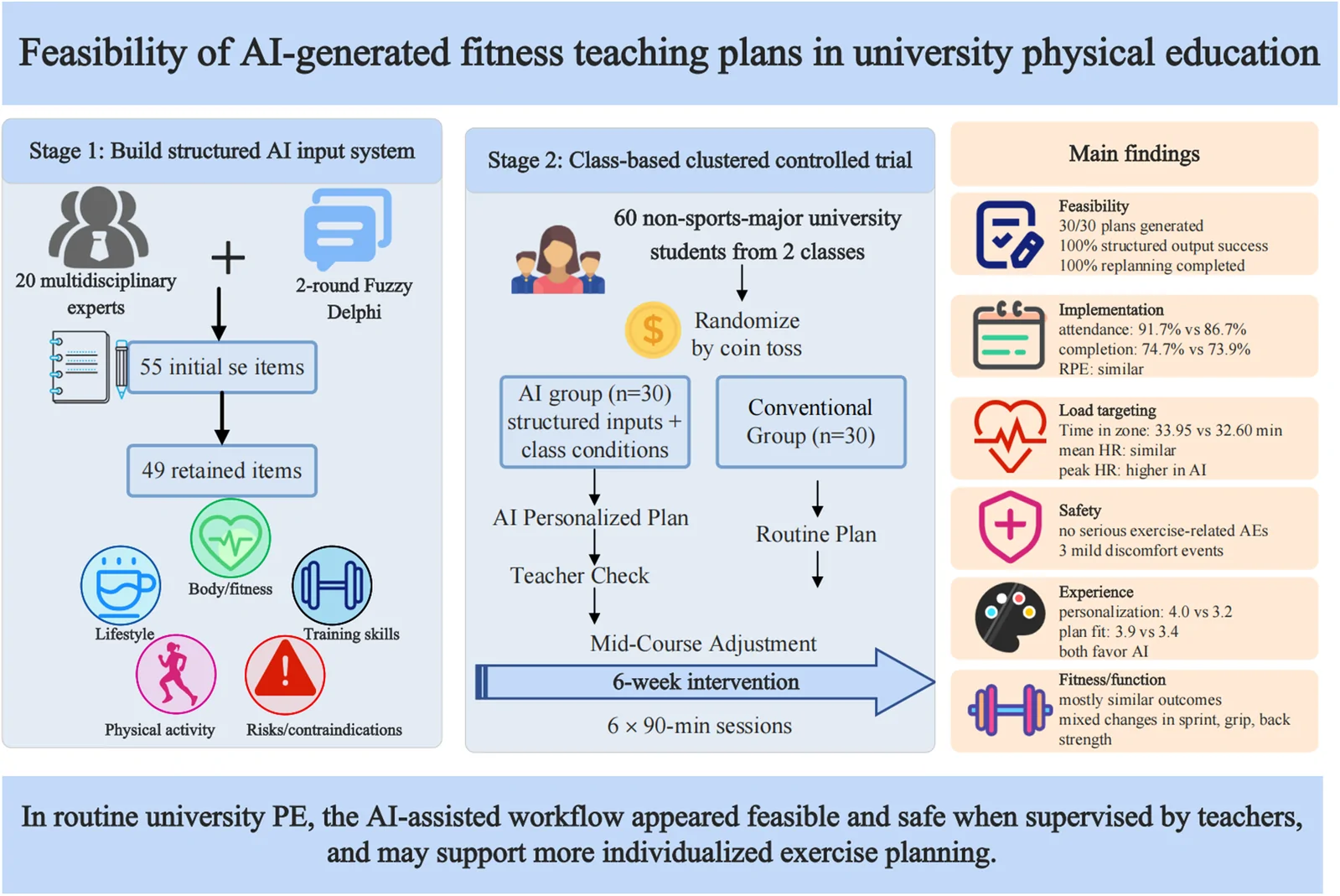
Infographic illustrates a twostage study on AI-generated fitness teaching plans for university physical education. Stage one developed a structured AI input system using 20 experts and Delphi method, leading to 49 retained items covering lifestyle, body/fitness, training skills, physical activity, and risks. Stage two involved a six-week, class-based trial with sixty students split into AI and conventional groups, evaluating personalized plans versus routine plans. Main findings highlight feasibility, high implementation and attendance rates, similar safety and load targeting, increased experience ratings for AI, and mostly similar fitness outcomes, supporting individualized exercise planning under teacher supervision.

## 1 Introduction

University public physical education courses provide an important opportunity to promote physical activity and improve physical fitness among college students (1). However, individualized instruction is difficult to implement in routine teaching because of limited class time, fixed facilities, and substantial heterogeneity in students' fitness levels, exercise experience, and health-related characteristics (2–4). Individualized exercise prescription has been widely advocated because it allows training to be tailored to individual needs and goals (5). In university physical education, however, developing personalized training plans for large classes is challenging and often relies heavily on instructor judgment, which may reduce consistency and reproducibility.

Recent advances in large language model–based generative artificial intelligence (AI) offer a potential low-cost approach to support individualized instructional planning. Tools such as ChatGPT can process structured inputs and generate personalized exercise recommendations efficiently and at scale (6). However, existing evidence has focused mainly on medical care (7, 8), rehabilitation (9), nursing (10), medical education (11–13), and broader digital health settings (14–16) Importantly, the quality and safety of AI-generated exercise plans depend not only on the information provided to the model but also on appropriate instructor oversight. Therefore, a structured input framework is needed to support standardized, auditable, and teacher-supervised use of AI-assisted exercise planning.

This study aimed to develop a structured input framework (17, 18) and evaluate the feasibility, safety, acceptability, and preliminary outcomes of teacher-supervised ChatGPT-assisted fitness instruction in university physical education (19, 20).

## 2 Methods

### 2.1 Study design

This study was conducted in two distinct phases.

Phase I employed the Fuzzy Delphi Method (FDM) (21) to construct a comprehensive input variable framework for generating AI-driven personalized instructional plans using ChatGPT(OpenAI) in university fitness classrooms.

Phase II used a class-based cluster pilot trial, with intact classes as the unit of allocation, to evaluate the feasibility, implementability, safety, acceptability, and preliminary effects of ChatGPT-assisted fitness instruction compared with conventional instruction in university physical education courses (20). The randomization was performed at the level of intact natural classes to minimize intervention contamination among students within the same class and to preserve the ecological validity of the instructional setting (22–24). The intervention spanned 6 weeks, consisting of six 90-minute sessions. Comprehensive measurements and evaluations were conducted both before and after the intervention period (25).

### 2.2 Phase I: construction of the input variable framework for AI-driven fitness instruction

#### 2.2.1 Expert panel selection

A purposive sampling strategy was used to recruit experts from diverse fields, including sports training, physical education, clinical medicine, clinical psychology, and artificial intelligence. A total of 20 experts were invited to participate, representing universities, public healthcare institutions, private fitness organizations, and research institutes.

#### 2.2.2 Development of the initial item pool

Based on a systematic literature review, classroom instructional needs, and practical application scenarios for AI-Driven personalized plans, the research team developed an initial pool of 55 items. These items were categorized into five dimensions:

1. Demographics and Lifestyle;
2. Physical Activity Levels;
3. Anthropometric Measures and Physical Fitness Test Scores;
4. Training Skills and Fundamental Capabilities;
5. Risk Factors and Contraindications.

#### 2.2.3 Expert consultation and fuzzy Delphi analysis

Experts rated the importance of each item using a 5-point linguistic scale. These linguistic assessments were converted into triangular fuzzy numbers (TFNs) denoted as (L, M, U) and aggregated across the panel. Consensus was evaluated using the vertex method to calculate the threshold distance (*d*) (26). Additionally, expert consensus rates and defuzzified scores were computed to guide item selection.

Referencing established FDM protocols (27), the criteria for retaining items in the first round were defined as: (1) Threshold distance (*d*) ≤0.20; (2) Expert consensus rate ≥75%; (3) Defuzzified score ≥0.50.

Items that did not meet the consensus criteria were reviewed and revised by the research team based on qualitative feedback from the experts. Revisions included ‘*removing items with weak relevance*’, ‘merging overlapping medical indicators’, and ‘clarifying the distinction between instructor-reported’ and ‘student-reported variables’. A second-round questionnaire was subsequently administered to finalize the ‘AI Personalized Instruction Input Framework’ tailored for university fitness classrooms.

#### 2.2.4 Assessment of expert authority

To evaluate the credibility of the expert consultation, the Expert Authority Coefficient (Cr) was calculated for each participant based on the Self-assessed Familiarity Coefficient (Cs) and the Judgment Basis Coefficient (Ca), according to the following formula:

Cr=(Cs+Ca)/2

In accordance with previous Delphi studies, a Cr value of ≥0.70 was considered indicative of acceptable expert authority. However, experts with slightly lower Cr values were retained when they possessed complementary interdisciplinary expertise relevant to both physical education and AI-assisted instructional design. This approach was adopted to preserve the breadth of perspectives while maintaining the overall credibility of the expert panel.

### 2.3 Phase II: class-based pilot trial

#### 2.3.1 Participants

The study population consisted of non-sports-major undergraduates enrolled in elective fitness courses within the public physical education program at a university. Participants were recruited from diverse majors, including Accounting, International Trade, Finance, Law, and Computer Science.

Inclusion criteria:

1. Enrolled in the public physical education fitness course;
2. Within the typical age range for university students;
3. Medically cleared to participate in regular physical training.

Exclusion criteria:

1. Presence of explicit contraindications to exercise;
2. Recent severe musculoskeletal injuries or acute illnesses;
3. Inability to complete baseline or outcome measurements;
4. Anticipated inability to attend classes regularly during the study period.

All experimental procedures followed the principles of the Helsinki Declaration and were approved by the Institutional Ethical Committee of Guangzhou College of Commerce (2025-Ethical Review-002) and was prospectively registered with the Chinese Clinical Trial Registry (ChiCTR2500107730) on 08/18/2025. This study is part of the registered study. The participants voluntarily signed the informed consent form after being fully aware of the experimental procedure and precautions. This study was reported in accordance with the Consolidated Standards of Reporting Trials (CONSORT) guidelines.

#### 2.3.2 Randomization and allocation

A cluster pilot trial was employed, with intact natural classes serving as the unit of allocation. Two classes (Class A: *n* = 36; Class B: *n* = 35) were initially screened. After applying the inclusion and exclusion criteria, 30 students from each class were eligible and provided written informed consent (Figure 1).

**Figure 1:**
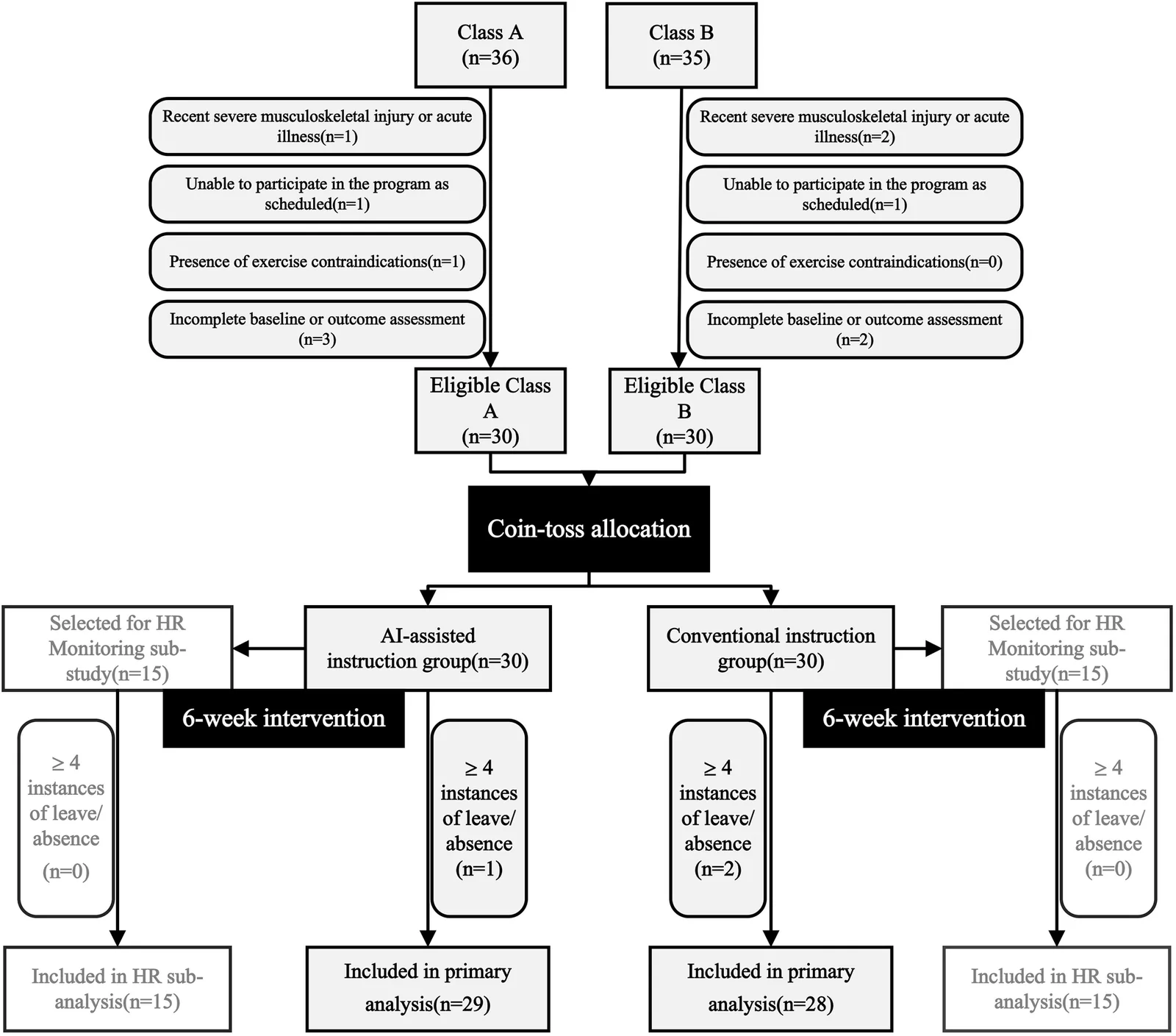
Participant flow diagram of the cluster pilot trial and nested heart-rate monitoring sub-study.

To minimize contamination within the same teaching setting, the two intact classes were assigned by coin toss to either the AI-assisted instruction group or the conventional instruction group.

#### 2.3.3 Intervention protocols

##### 2.3.3.1 AI-assisted instruction group

Personalized training plans were generated by ChatGPT(OpenAI) based on the structured input framework developed in Phase I. Inputs included student characteristics (demographics, physical activity level, anthropometrics, fitness performance, skills, and risk factors) and classroom conditions (facility availability and class size).

The AI-generated plans specified training objectives, exercise selection, volume, intensity, and safety precautions. All plans were reviewed by certified physical education instructors before implementation using predefined safety and feasibility criteria. Plans requiring modification were revised before use.

To support dynamic personalization, a mid-intervention replanning session was conducted after Session 3 using participant feedback and implementation records.

##### 2.3.3.2 Conventional instruction group

The conventional instruction group followed training plans designed by experienced instructors according to standard university physical education curricula. Exercise content, progression, and intensity adjustments were determined using routine pedagogical practice and overall class performance. Sessions were delivered by the same level of certified instructors as the AI-assisted group.

#### 2.3.4 Implementation procedure

The intervention was conducted from September to November 2025 at Guangzhou College of Commerce. Both groups completed a 6-week program consisting of six 90-minute sessions.

Before each session, instructors provided standardized safety instructions. During training, instructors supervised exercise execution, recorded attendance and completion rates, and documented pain, discomfort, or protocol deviations. Exercise regressions were applied when necessary according to predefined safety rules. Because this was a parallel cluster pilot trial, no washout period was required.

### 2.4 Outcome measures

#### 2.4.1 Phase I outcomes

1. Outcomes for the Fuzzy Delphi phase included:
2. Expert demographic characteristics;
3. Expert Authority Coefficient (Cr);
4. Threshold distance (*d*) for each item;
5. Expert consensus rate;
6. Defuzzified score;

Final item retention status and priority rankings across dimensions.

#### 2.4.2 Primary outcomes (phase II)

Primary outcomes focused on feasibility and implementability, including attendance frequency and rate, perfect attendance rate, session completion rate, Rating of Perceived Exertion (RPE), and heart rate (HR) measures. RPE was assessed using the Borg Category-Ratio 10 (CR10) scale (0 = “nothing at all”, 10 = “maximal exertion”). Due to limited device availability, HR data were collected from a subsample using wearable monitors (Domor HR monitors). The target HR zone was defined as 60%–80% of age-predicted maximum HR (220—age). Time in Zone (TIZ), calculated as cumulative minutes spent within the target range during the 50-minute training period, served as the primary HR indicator (28, 29).

Session completion rate was calculated as the proportion of completed training blocks relative to the planned volume (3 blocks × 2 rounds). All participants recorded completion rate and post-session RPE, whereas the HR-monitored subsample additionally recorded average HR, peak HR, and TIZ.

#### 2.4.3 AI system performance and audit metrics

Metrics to evaluate the operability and traceability of the AI system included:

1. Success rate of structured plan generation;
2. Frequency of generation interruptions triggered by contradictory inputs requiring clarification;
3. Adherence to the scheduled mid-intervention replanning;
4. Frequency of instructor-requested plan modifications prior to implementation.

#### 2.4.4 Safety

Safety was assessed through instructor review using predefined criteria, including appropriate exercise intensity, absence of contraindicated exercises, gradual progression, and feasibility within available facilities and equipment. Plans failing to meet any criterion were revised before implementation. Participant safety was additionally monitored through reports of pain, discomfort, excessive fatigue, and adverse events during training sessions.

## 3 Results

### 3.1 Part1

#### 3.1.1 Expert demographics and authority coefficients

A total of 20 experts from diverse backgrounds-including physical education instructors, medical practitioners, clinical psychologists, and artificial intelligence researchers-were recruited from Chinese universities, public healthcare institutions, and private sectors to participate in a two-round Delphi consultation. The panel composition was as follows: universities (*n* = 8), hospitals (*n* = 8), fitness institutions (*n* = 2), and research institutes (*n* = 2). The majority of experts possessed over 10 years of professional experience. Regarding educational attainment, 50% held a master's degree and 40% a doctoral degree. In terms of professional titles, 25% held senior positions (associate professor level or above), while 65% held intermediate titles.

The expert authority coefficient (Cr) was calculated based on self-assessed familiarity coefficient (Cs) and judgment criteria coefficient (Ca) using the formula Cr = (Cs + Ca)/2. The overall Cr for the panel was 0.82 ± 0.10, indicating a high level of authority and reliability among the experts regarding the “AI-driven fitness teaching input framework” (Table 1).

**Table 1:** Experts’ demography.

Title	YearsExpCat	OrgType	Cr	Position	Address
Senior	>10 Years	University	1	National First-Class Athlete, Sports Training Instructor (Specializing in Fitness)	Kaifeng, Henan
Intermediate	6–10 Years	University	1	Physical Health Promotion Instructor	Guangzhou, Guangdong
Intermediate	6–10 Years	University	1	Anatomy Instructor	Zhumadian, Henan
Intermediate	>10 Years	University	0.9	National First-Class Athlete, Sports Training Instructor (Specializing in Football/Soccer)	Baotou, Inner Mongolia
Intermediate	>10 Years	University	0.9	National First-Class Athlete, Sports Training Instructor (Specializing in Volleyball)	Qiqihar, Heilongjiang
Intermediate	6–10 Years	University	0.75	National Master of Sports, Sports Training Instructor (Specializing in Shuttlecock/Jianzi)	Guangzhou, Guangdong
Intermediate	6–10 Years	University	0.9	National First-Class Athlete, Sports Training Instructor (Specializing in Volleyball)	Guangzhou, Guangdong
Intermediate	>10 Years	University	0.9	National First-Class Athlete, Sports Training Instructor (Specializing in Table Tennis)	Kaifeng, Henan
Junior	6–10 Years	Fitness Institution	0.95	National First-Class Athlete, Sports Training Instructor (Specializing in Basketball)	Harbin, Heilongjiang
Junior	3–5 Years	Fitness Institution	0.85	National Second-Class Athlete, Fitness Specialist	Chaozhou, Guangdong
Intermediate	6–10 Years	Research Institute	0.75	AI Expert	Guangzhou, Guangdong
Intermediate	6–10 Years	Research Institute	0.75	AI Expert	Guangzhou, Guangdong
Intermediate	>10 Years	Hospital	0.85	TCM Physician, Health Management Center	Jinan, Shandong
Intermediate	>10 Years	Hospital	0.85	Internist, Exercise Prescription Specialist, Registered Dietitian (Health Management Center)	Yulin, Guangxi
Senior	>10 Years	Hospital	0.75	Cardiologist, Health Management Center	Weifang, Shandong
Intermediate	6–10 Years	Hospital	0.75	Exercise Prescription Specialist, Rehabilitation Technician	Guangzhou, Guangdong
Senior	>10 Years	Hospital	0.9	Rehabilitation Physician	Chaozhou, Guangdong
Senior	>10 Years	Hospital	0.6	Cardiologist, Health Management Center	Guangzhou, Guangdong
Intermediate	6–10 Years	Hospital	0.85	Endocrinologist, Health Management Center	Guangzhou, Guangdong
Senior	>10 Years	Hospital	0.8	Psychologist/Mental Health Physician, Health Management Center	Guangzhou, Guangdong

YearsExpCat, years of experience category; OrgType, organization type; Cr, the expert authority coefficient.

#### 3.1.2 Fuzzy Delphi consensus testing and item screening

The initial item pool comprised 55 items across five dimensions: Basic Information and Lifestyle, Physical Activity Level, Physical Indices and Fitness Test Performance, Training Skills and Fundamental Capabilities, and Risks and Contraindications. Following the first round of the fuzzy Delphi process, 19 items were identified for further review because their consensus indicators did not fully meet the prespecified criteria (Supplementary Table S1). Based on the quantitative results and expert feedback, revisions were made in four areas: removal of low-relevance items, consolidation of overlapping clinical indicators, optimization of data sources, and retention of key physical activity baselines. Items related to smoking status, alcohol consumption, and reaction time were excluded because they were considered to have limited relevance to AI-driven fitness prescription. Liver and renal function markers were simplified into a single binary item indicating a physician-reported history of abnormal liver or kidney function. Objective fitness measures and classroom logistics were reassigned from student self-report to instructor report to improve accuracy and reduce recall bias. In contrast, core IPAQ-derived indicators, including walking duration, walking frequency, and average daily steps, were retained because they were regarded as essential for characterizing baseline activity and informing individualized plan generation. Overall, these revisions enhanced the feasibility, clarity, and practical applicability of the final input framework.

In the second round, 13 experts responded. All four proposed modifications were accepted (Supplementary Table S2). The final item pool consisted of 49 items, including 34 student-reported items and 15 instructor-reported items. Overall, all items met the threshold distance criterion (*d* ≤ 0.20), and 96.4% achieved the predefined consensus rate of ≥75%, indicating a high level of agreement among experts regarding the input information set required for AI-driven fitness teaching. For a small number of borderline items, such as those with consensus rates close to 75% or defuzzified scores near 0.2, the research team reviewed the quantitative findings alongside the experts' open-ended feedback. These items were retained only if they were considered important for maintaining the comprehensiveness of the framework while not imposing unnecessary data-collection burden.

#### 3.1.3 Dimension-specific item retention and prioritization

Across dimensions, experts tended to prioritize items that were directly relevant to the safety and feasibility of individualized in-class exercise prescription. In the Risks and Contraindications dimension, items related to pain/injury history, PAR-Q red flags, medication/allergies, and physician-imposed restrictions showed the strongest consensus. In Training Skills and Fundamental Capabilities, movement proficiency, training history, and recent training frequency/duration were prioritized. In Physical Activity Level, sedentary time, daily step count, and exercise barriers/goals were considered important. In Physical Indices and Fitness Test Performance, BMI, resting heart rate, and key fitness test results were retained as core indicators for exercise intensity stratification. In Basic Information and Lifestyle, sleep quality was identified as an important factor related to fatigue and training response.

#### 3.1.4 Formation of the final input framework

In conclusion, this study employed a two-round Fuzzy Delphi method with a multidisciplinary expert panel to establish an “AI-Driven Individualized Teaching Input Framework” tailored for university fitness classrooms. By applying a triple-criteria validation system-comprising threshold distance (*d*), expert consensus rate, and defuzzified scores-we ensured the robustness and auditability of the item selection process. This framework provides a solid foundation of content validity for subsequent structured prompt engineering and empirical classroom validation (Supplementary Appendix 1).

### 3.2 Part 2

#### 3.2.1 Participant flow and follow-up completion

The intervention was implemented at the class level within elective physical education fitness courses. Participants were non-sports-major university students majoring in accounting, international trade, finance, law, and computer science. Each class consisted of 30 subjects who meet the inclusion and exclusion criteria and voluntarily participate in this study. Classes were randomized into two groups using a coin-toss method: the AI-assisted instruction group (*n* = 30) and the conventional instruction group (*n* = 30). Upon completion of the 6-week intervention, 29 participants in the AI-assisted instruction group and 28 in the Conventional group completed all interventions and outcome measurements, yielding completion rates of 96.7% and 93.3%, respectively. Three participants discontinued follow-up primarily due to leave of absence or excessive absenteeism (≥4 missed sessions) caused by university activities (Figure 1).

#### 3.2.2 Baseline characteristics

Baseline demographic characteristics, physical activity levels, health screening results, and physical/functional test scores for both groups are presented in Table 2. Overall, no significant differences were observed between the AI and Conventional group regarding age, gender distribution, baseline total fitness scores, or key physical performance metrics, indicating good baseline comparability. Specifically, the mean ages were 19.48 ± 0.86 years (AI) and 19.76 ± 1.92 years (Conventional). The proportion of male participants was 60% in both groups. Baseline back strength scores were 82.97 ± 38.03 and 87.52 ± 34.56 for the AI and Conventional groups, respectively.

**Table 2:** Baseline characteristics of the study participants.

Variable	Overall	Group AI	Group Con	*P*_value
Age, year	19.62 ± 1.41	19.48 ± 0.57	19.76 ± 1.92	0.972
Body mass index, kg/m^2^	21.41 ± 3.59	20.95 ± 3.57	21.85 ± 3.62	0.299
Fitness assessment results, score	71.72 ± 9.05	71.68 ± 10.49	71.76 ± 7.53	0.836
Systolic blood pressure, mmHg	127.40 ± 18.60	126.43 ± 20.13	128.37 ± 17.22	0.663
Diastolic blood pressure, mmHg	71.60 ± 9.90	70.93 ± 11.24	72.27 ± 8.49	0.446
Heart rate, bpm	83.95 ± 14.63	84.27 ± 16.69	83.62 ± 12.43	0.82
Back strength, kg	85.20 ± 36.13	82.97 ± 38.03	87.52 ± 34.56	0.693
Left grip, kg	27.73 ± 10.64	27.93 ± 10.15	27.53 ± 7.82	0.871
Right grip, kg	36.53 ± 38.00	40.68 ± 53.18	32.51 ± 11.13	0.733
Gender, male	36 (60.0%)	18 (60.0%)	18 (60.0%)	1
Smoke, yes	3 (5.2%)	3 (10.3%)	0 (0.0%)	0.237
Alcohol, yes	4 (6.9%)	2 (6.9%)	2 (6.9%)	1
IPAQ, lowlevel	11 (19.6%)	5 (18.5%)	6 (20.7%)	0.669
IPAQ, midlevel	32 (57.1%)	17 (63.0%)	15 (51.7%)	
IPAQ, highlevel	13 (23.2%)	5 (18.5%)	8 (27.6%)	

IPAQ, international physical activity questionnaire; Group Con, conventional group; Group AI, AI-assisted group.

#### 3.2.3 Intervention implementation and adherence

The mean attendance was 5.49 ± 1.06 sessions (attendance rate: 91.7%) for the AI-assisted instruction group and 5.20 ± 1.19 sessions (attendance rate: 86.7%) for the Conventional group, with no significant difference between groups (*P* = 0.315). The average completion rate across the six sessions was 74.74 ± 18.03% for the AI-assisted instruction group and 73.90 ± 17.42% for the Conventional instruction group (*P* = 0.682). Regarding rating of perceived exertion (RPE), the mean RPE score was 5.86 ± 0.77 in the AI-assisted instruction group and 5.71 ± 0.74 in the conventional instruction group (*P* = 0.068). (Supplementary Table S3).

In the HR sub-study, 30 participants were included (AI-assisted instruction group: *n* = 15; Conventional instruction group: *n* = 15). A total of 87 and 82 analyzable training sessions were obtained from the AI-assisted instruction and Conventional instruction groups, respectively; missing sessions were mainly attributable to student leave. The target HR zone was defined as 60%–80% of age-predicted maximum HR. Time in Zone (TIZ) was calculated as the cumulative time spent within this target range during the 50-minute main training period. Results showed that the AI-assisted instruction group spent significantly more time in the effective training zone compared to the Conventional instruction group (33.95 ± 4.33 vs. 32.60 ± 3.74 min; *P* = 0.033). While no significant difference was found in mean HR (112.1 ± 7.9 vs. 110.9 ± 7.5 bpm; *P* = 0.311), the AI-assisted instruction group exhibited a significantly higher maximum HR (164.8 ± 19.8 vs. 153.9 ± 13.3 bpm; *P* < 0.001) (Supplementary Table S3).

#### 3.2.4 AI plan generation success and auditability

The AI system successfully generated individualized training plans for all 30 participants assigned to the AI-assisted instruction group, with all outputs conforming to the prespecified structured template. In accordance with the protocol, all participants underwent one mid-intervention replanning after Session 3, which was completed without interruption in all cases. No additional clarification was required because of contradictory input information. The Conventional instruction group followed the standard progression-based adjustment of training intensity (Supplementary Appendix 2).

#### 3.2.5 Safety and intervention fidelity

No serious exercise-related adverse events occurred during the intervention. Three minor adverse events were recorded, consisting of muscle soreness and transient dizziness; none progressed to a moderate or severe level, and no participant withdrew because of an adverse event.

Pain scores remained low in both groups. The proportion of sessions with pain scores ≥5 (10-point scale) was 2.4% (4/165 sessions) in the AI-assisted instruction group and 1.9% (3/156 sessions) in the conventional instruction group. All reported pain events were mild and consistent with transient muscle soreness.

Regarding intervention fidelity, all AI-generated plans underwent instructor review before implementation as a safety assurance measure. No plans required modification following review, and all were approved for implementation without revision.

The execution rate for pre-class safety cues (e.g., “exhale during exertion” and “avoid the Valsalva maneuver”) and stop-rule instructions was 100% in both groups. Instructor monitoring fidelity, defined *a priori* as completion of at least two full circuit patrols during the main training period, was also 100% in both groups (6/6 sessions each), corresponding to an overall intervention fidelity rating of “High.”

Across all 12 scheduled group sessions (6 per group), three minor protocol deviations were recorded (2 in the AI-assisted instruction group and 1 in the conventional instruction group). All were safety-related downward adjustments made in response to participant-reported pain or fatigue. According to the protocol, these deviations were documented as permissible modifications rather than fidelity violations.

#### 3.2.6 Acceptability and perceived appropriateness

The AI-assisted instruction group scored significantly higher than the Conventional instruction group on perceived personalization and fit/appropriateness. On a 5-point scale, the mean perceived personalization score was 4.0 ± 0.7 in the AI-assisted instruction group vs. 3.2 ± 1.0 in the Conventional instruction group (*P* = 0.001). Similarly, the mean fit score was 3.9 ± 0.7 in the AI-assisted instruction group compared with 3.4 ± 0.8 in the Conventional instruction group (*P* = 0.020). These results indicate more favorable perceptions of personalization and appropriateness in the AI-assisted instruction group (Supplementary Table S4).

#### 3.2.7 Changes in physical and functional metrics

ANCOVA was conducted using post-test values as the dependent variables, group as the independent variable, and corresponding baseline values as covariates. After baseline adjustment, no significant between-group differences were found for most physical and functional outcomes (all *P* > 0.05). Only three outcomes showed significant group differences: 50-m sprint, left-hand grip strength, and back strength (Table 3).

**Table 3:** Effects of the intervention on physical fitness parameters.

Variable	Group Con	Group AI	beta_AI_minus_Con	SE	95% CI	*P*_value
Vital capacity, mL	28	27	24.164	230.778	[−438.927, 487.254]	0.917
Sit-and-reach, cm	28	27	−2.168	2.259	[−6.701, 2.365]	0.342
50-meter sprint, s	28	26	−0.58	0.271	[−1.124, −0.036]	0.037
Standing long jump, m	28	26	12.87	12.514	[−12.253, 37.993]	0.309
800-meter run, min	11	11	0.153	0.32	[−0.517, 0.824]	0.637
1,000-meter run, min	17	15	0.452	0.465	[−0.499, 1.402]	0.339
1-minute sit-up, times	11	11	−1.846	2.076	[−6.192, 2.499]	0.385
Pull-up, times	17	15	3.113	1.869	[−0.71, 6.935]	0.107
Back strength, kgf	27	28	−27.35	6.85	[−41.095, −13.605]	<0.001
Left hand grip strength, kgf	28	28	9.745	2.664	[4.403, 15.088]	<0.001
Right hand grip strength, kgf	28	28	−0.344	10.63	[−21.665, 20.977]	0.974
Physical fitness test score	28	28	−1.179	2.282	[−5.756, 3.398]	0.607

Kgf, kilogram-force; Group Con, Conventional group; Group AI, AI-assisted group.

Compared with the Conventional instruction group, the AI-assisted instruction group demonstrated a lower post-test time in the 50-m sprint (*β* = −0.580, 95% CI: −1.124 to −0.036, *P* = 0.037) and a higher post-test value for left-hand grip strength (*β* = 9.745, 95% CI: 4.403–15.088, *P* < 0.001), but a lower post-test value for back strength (*β* = −27.350, 95% CI: −41.095 to −13.605, *P* < 0.001).

## 4 Discussion

This study suggests that ChatGPT-assisted individualized fitness instruction is feasible in university physical education when implemented within a structured and teacher-supervised workflow. Under routine teaching conditions, plan generation was stable, mid-course replanning was achievable, and no obvious short-term additional safety concerns emerged during teacher review or classroom delivery (30, 31). The main implication is not that a large language model can independently prescribe exercise, but that it can be incorporated into a practical instructional workflow including structured input, bounded prompting, teacher review, classroom implementation, and protocol-based adjustment (32–34).

A key strength of the study is its real-world setting. The intervention was delivered in routine public physical education classes involving non-sports-major students, fixed class schedules, limited equipment, and usual teaching constraints. Within this context, attendance, completion, and perceived exertion were similar between groups, suggesting that AI-assisted planning did not disrupt routine instruction or reduce participation. This supports the practical relevance of the approach for everyday teaching settings rather than idealized experimental conditions (33).

The clearest between-group difference was seen in students' subjective perceptions. The AI-assisted instruction group reported better perceived individualization and better fit of the training plan. In university physical education, this is meaningful because students may respond not only to the absolute training load, but also to whether the program feels appropriate for their ability and needs. In large classes, where true one-to-one adjustment is difficult, perceived fit may be an important early indicator of instructional quality and implementation success (35, 36). The present findings therefore suggest that one near-term value of AI-assisted planning may lie in improving how well students feel the program matches them.

An unexpected finding was the significantly lower post-intervention back strength in the AI-assisted group. However, this isolated result was not accompanied by consistent differences in other physical fitness outcomes and may reflect random or class-level variation given the pilot design, small sample size, and limited number of clusters (37, 38). In contrast, perceived personalization and plan fit were consistently higher in the AI-assisted group, representing the most robust advantage of the intervention. This finding suggests that the primary value of generative AI may lie in enhancing perceived instructional relevance and individual fit rather than short-term physical fitness outcomes. However, this benefit should also be interpreted cautiously, as it may have been partly influenced by novelty effects associated with AI-assisted planning. Whether such perceptions persist over longer implementation periods warrants further investigation.

Safety should be interpreted cautiously. No serious adverse events occurred, and all reported discomfort events were mild. However, all AI-generated plans were reviewed by teachers before use, and teachers retained full authority to adjust implementation during class. The observed safety profile should therefore be understood as a property of the supervised workflow rather than of the model alone. These findings support a teacher-in-the-loop approach in which AI assists with plan structuring and updating, while instructors remain responsible for screening, judgment, technique correction, and real-time monitoring.

Consistent with prior research, Iuliano et al. reported that non-traditional instructional formats can achieve comparable student satisfaction while still eliciting preference for traditional face-to-face teaching in direct comparisons (39). Tao et al. further highlight that satisfaction in university physical education is multidimensional, shaped by instructional organization, teaching quality, and curriculum structure (40). In this context, the present AI-assisted workflow primarily modifies organizational and structural aspects of instruction, suggesting that observed acceptance reflects changes in specific satisfaction dimensions rather than a single global evaluation of teaching effectiveness.

Several limitations should be acknowledged. First, although a class-based clustered design was used, the intervention involved only two intact classes, with one class allocated to each condition. Consequently, observed between-group differences may partly reflect class-level characteristics rather than intervention effects, and the effectiveness findings should therefore be interpreted cautiously as exploratory rather than confirmatory. Second, the sample size was modest and the intervention period was short. Third, heart-rate monitoring was available only in a subsample. Fourth, the experience-related outcomes were based on post-class ratings and may have been influenced by novelty effects. Finally, because only two clusters were included, intracluster correlation coefficients could not be estimated reliably. Accordingly, this study should be interpreted as a cluster pilot feasibility trial rather than a definitive effectiveness study Accordingly, this study should be interpreted as a cluster pilot feasibility trial rather than a definitive effectiveness study (41).

Despite these limitations, the study provides an initial practical model for the use of generative AI in university physical education. The workflow tested here—structured input, bounded prompting, teacher review, classroom delivery, and mid-course replanning—appeared workable under routine teaching constraints. Future research should evaluate this workflow in multi-site cluster-randomized trials involving substantially more class clusters to permit estimation of cluster effects and intracluster correlation coefficients. Intervention periods of at least one academic semester may be needed to determine whether improvements in perceived personalization translate into sustained changes in adherence, training quality, and physical fitness. Candidate primary outcomes for a definitive trial could include perceived personalization, adherence, time in target heart-rate zone, and selected fitness indicators measured using standardized protocols (42).

In conclusion, ChatGPT-assisted individualized fitness instruction was feasible to implement in university physical education when embedded within a structured and teacher-supervised workflow. In this pilot study, its main advantage was not broad short-term superiority in physical fitness outcomes, but a better perceived match between the training plan and the student. At this stage, generative AI should be viewed as a supportive tool for enhancing scalable personalization in routine physical education practice rather than as a substitute for professional judgment or in-class supervision.

## Data Availability

The original contributions presented in the study are included in the article/Supplementary Material, further inquiries can be directed to the corresponding authors.
