# Supplementary material for: Feasibility of a teacher-supervised ChatGPT-assisted workflow for individualized exercise planning in university physical education: a two-phase study using a fuzzy Delphi process and a cluster pilot trial
Source: Front Sports Act Living. 2026 Sep 25;8:1896856. doi: 10.3389/fspor.2026.1896856 (PMC13649966; doi:10.3389/fspor.2026.1896856)
Supplement: Supplementary file 1 [file Datasheet1.docx]

**Table S1.Results of the Fuzzy Delphi Method Analysis for AI-exercise Items for the first time.**

| **Dimension** | **Item** | **Expert consensus rate** | **Defuzzified score** | **Threshold value, d** | **Expert consensus** |
| --- | --- | --- | --- | --- | --- |
| Dimension 1: Basic Information & Lifestyle | Age | 0.85 | 0.733 | 0.075 | Accepted |
| Dimension 1: Basic Information & Lifestyle | Gender | 0.6 | 0.625 | 0.097 | Discuss |
| Dimension 1: Basic Information & Lifestyle | Class Frequency | 0.95 | 0.788 | 0.054 | Accepted |
| Dimension 1: Basic Information & Lifestyle | Duration per Session | 0.9 | 0.767 | 0.053 | Accepted |
| Dimension 1: Basic Information & Lifestyle | Training Cycle / Periodization | 0.95 | 0.813 | 0.065 | Accepted |
| Dimension 1: Basic Information & Lifestyle | Equipment / Venue | 0.65 | 0.667 | 0.083 | Discuss |
| Dimension 1: Basic Information & Lifestyle | Class Size | 0.6 | 0.646 | 0.083 | Discuss |
| Dimension 1: Basic Information & Lifestyle | Smoking in Last 3 Days | 0.55 | 0.638 | 0.113 | Discuss |
| Dimension 1: Basic Information & Lifestyle | Alcohol Consumption in Last 3 Days | 0.6 | 0.658 | 0.116 | Discuss |
| Dimension 1: Basic Information & Lifestyle | Sleep Quality | 0.95 | 0.813 | 0.065 | Accepted |
| Dimension 1: Basic Information & Lifestyle | Pre-Exercise Nutrition / Intake | 0.75 | 0.721 | 0.069 | Accepted |
| Dimension 1: Basic Information & Lifestyle | Stress Score | 0.8 | 0.742 | 0.066 | Accepted |
| Dimension 2: Physical Activity Level | Vigorous Activity Frequency | 0.8 | 0.733 | 0.061 | Accepted |
| Dimension 2: Physical Activity Level | Vigorous Activity Duration | 0.9 | 0.758 | 0.046 | Accepted |
| Dimension 2: Physical Activity Level | Moderate Activity Frequency | 0.85 | 0.746 | 0.053 | Accepted |
| Dimension 2: Physical Activity Level | Moderate Activity Duration | 0.8 | 0.713 | 0.079 | Accepted |
| Dimension 2: Physical Activity Level | Walking Frequency | 0.55 | 0.604 | 0.124 | Discuss |
| Dimension 2: Physical Activity Level | Walking Duration | 0.55 | 0.604 | 0.124 | Discuss |
| Dimension 2: Physical Activity Level | Average Daily Steps | 0.6 | 0.608 | 0.119 | Discuss |
| Dimension 2: Physical Activity Level | Sedentary Time | 0.85 | 0.717 | 0.066 | Accepted |
| Dimension 2: Physical Activity Level | Intensity Preference | 0.8 | 0.729 | 0.087 | Accepted |
| Dimension 2: Physical Activity Level | Exercise Barriers / Limitations | 1 | 0.817 | 0.057 | Accepted |
| Dimension 2: Physical Activity Level | Exercise Goal / Purpose | 0.95 | 0.792 | 0.069 | Accepted |
| Dimension 3: Physical Metrics & Fitness Test Results | Height | 0.35 | 0.554 | 0.1 | Discuss |
| Dimension 3: Physical Metrics & Fitness Test Results | Weight | 0.8 | 0.738 | 0.08 | Accepted |
| Dimension 3: Physical Metrics & Fitness Test Results | Body Mass Index | 0.8 | 0.721 | 0.083 | Accepted |
| Dimension 3: Physical Metrics & Fitness Test Results | Blood Pressure | 0.95 | 0.804 | 0.062 | Accepted |
| Dimension 3: Physical Metrics & Fitness Test Results | Resting Heart Rate | 0.8 | 0.721 | 0.071 | Accepted |
| Dimension 3: Physical Metrics & Fitness Test Results | Fasting Blood Glucose (FBG) | 0.9 | 0.75 | 0.039 | Accepted |
| Dimension 3: Physical Metrics & Fitness Test Results | Alanine Aminotransferase (ALT) | 0.65 | 0.671 | 0.07 | Discuss |
| Dimension 3: Physical Metrics & Fitness Test Results | Aspartate Aminotransferase (AST) | 0.7 | 0.683 | 0.064 | Discuss |
| Dimension 3: Physical Metrics & Fitness Test Results | Creatinine (Cr) | 0.85 | 0.738 | 0.047 | Accepted |
| Dimension 3: Physical Metrics & Fitness Test Results | Uric Acid (UA) | 0.65 | 0.667 | 0.094 | Discuss |
| Dimension 3: Physical Metrics & Fitness Test Results | Total Fitness Test Score | 0.8 | 0.7 | 0.09 | Accepted |
| Dimension 3: Physical Metrics & Fitness Test Results | Vital Capacity | 0.7 | 0.658 | 0.095 | Discuss |
| Dimension 3: Physical Metrics & Fitness Test Results | 50-Meter Sprint | 0.6 | 0.621 | 0.11 | Discuss |
| Dimension 3: Physical Metrics & Fitness Test Results | Standing Long Jump | 0.55 | 0.608 | 0.111 | Discuss |
| Dimension 3: Physical Metrics & Fitness Test Results | Sit-and-Reach Test | 0.55 | 0.633 | 0.095 | Discuss |
| Dimension 3: Physical Metrics & Fitness Test Results | 1000-Meter Run | 0.6 | 0.646 | 0.093 | Discuss |
| Dimension 3: Physical Metrics & Fitness Test Results | Pull-Ups | 0.55 | 0.642 | 0.1 | Discuss |
| Dimension 3: Physical Metrics & Fitness Test Results | Reaction Time | 0.65 | 0.679 | 0.074 | Discuss |
| Dimension 3: Physical Metrics & Fitness Test Results | Back Strength | 0.8 | 0.75 | 0.072 | Accepted |
| Dimension 3: Physical Metrics & Fitness Test Results | Grip Strength (Left) | 0.55 | 0.671 | 0.092 | Discuss |
| Dimension 3: Physical Metrics & Fitness Test Results | Grip Strength (Right) | 0.55 | 0.671 | 0.092 | Discuss |
| Dimension 3: Physical Metrics & Fitness Test Results | One-Leg Stand with Eyes Closed | 0.7 | 0.7 | 0.072 | Accepted |
| Dimension 4: Training Skills & Fundamental Capabilities | Mastery of the "Big Three" Techniques | 0.75 | 0.729 | 0.074 | Accepted |
| Dimension 4: Training Skills & Fundamental Capabilities | Years of Training Experience | 0.75 | 0.721 | 0.069 | Accepted |
| Dimension 4: Training Skills & Fundamental Capabilities | Movement Proficiency / Skill Level | 0.85 | 0.771 | 0.07 | Accepted |
| Dimension 4: Training Skills & Fundamental Capabilities | Training Frequency (Last Month) | 0.95 | 0.779 | 0.048 | Accepted |
| Dimension 4: Training Skills & Fundamental Capabilities | Duration per Session (Last Month) | 0.85 | 0.746 | 0.053 | Accepted |
| Dimension 4: Training Skills & Fundamental Capabilities | Proposed Intensity for Core Exercises | 0.95 | 0.804 | 0.062 | Accepted |
| Dimension 5: Risks & Contraindications | History of Pain / Injuries | 1 | 0.842 | 0.058 | Accepted |
| Dimension 5: Risks & Contraindications | PAR-Q+ Red Flags | 0.9 | 0.75 | 0.039 | Accepted |
| Dimension 5: Risks & Contraindications | Chronic Diseases / Medications / Allergies | 0.8 | 0.721 | 0.083 | Accepted |
| Dimension 5: Risks & Contraindications | Medical Restrictions on Exercise | 0.95 | 0.804 | 0.062 | Accepted |

**Table S2.Results of the Fuzzy Delphi Method Analysis for AI-exercise Items for the second time.**

| **Item** | **Expert consensus rate** | **Defuzzified score** | **Threshold value, d** | **Expert consensus** |
| --- | --- | --- | --- | --- |
| Upon evaluation, the items regarding smoking, alcohol consumption, and reaction time were found to have weak correlation with the formulation of AI fitness prescriptions; therefore, they are proposed for deletion. | 0.846 | 0.763 | 0.115 | Accepted |
| Liver function indicators (ALT, AST) and kidney function indicators (UA, Cr) are proposed to be merged and categorized under a single item: "History of Physician-Indicated Liver or Kidney Abnormalities," to simplify data collection. | 0.923 | 0.75 | 0.101 | Accepted |
| Physical fitness test results and classroom information are to be completed uniformly by the instructors. | 0.923 | 0.769 | 0.084 | Accepted |
| Walking duration, frequency, and average daily steps are core items of the IPAQ and are proposed to be retained. | 0.923 | 0.821 | 0.121 | Accepted |

ALT: Alanine Aminotransferase, AST: Aspartate Aminotransferase, UA: Uric Acid, Cr: Creatinine, IPAQ: International Physical Activity Questionnaire

**Table S3.Intervention Implementation Characteristics and Adherence.**

| **Characteristics and Adherence** | **Group AI** | **Group Con** | **P_value** |
| --- | --- | --- | --- |
| Average Attendance Count per Person, times | 5.48+1.06 | 5.20+1.19 | 0.315 |
| Average Completion Rate per Person, % | 74.74+18.03 | 73.9+17.42 | 0.682 |
| Rating of Perceived Exertion, score | 3.86+0.77 | 3.71+0.74 | 0.068 |
| Attendance Rate, % | 91.7+2.5 | 86.6+31.2 | 0.209 |
| Sub-study |  |  |  |
| Effective Main Training Time, min | 33.95+4.33 | 32.60+3.74 | 0.033 |
| Average Heart Rate, bpm | 112.1+7.9 | 110.9+7.5 | 0.311 |
| Maximum Heart Rate, bpm | 164.8+19.8 | 153.9+13.3 | ＜0.001 |

Group Con: Conventional group; Group AI: AI-assisted group

**Table S4.Personalization and Adaptability in Physical Education Curriculum Design.**

| **Personalization and Adaptability** | **Group AI** | **Group Con** | **P_value** |
| --- | --- | --- | --- |
| Personalization | 4.0+0.7 | 3.2+1.0 | 0.001 |
| Adaptability | 3.9+0.7 | 3.4+0.8 | 0.02 |

Group Con: Conventional group; Group AI: AI-assisted group

**Supplementary Appendix 1.Structured Prompt for Generating Individualized University Fitness Class Teaching Plans.**

prompt_version: P1.0-GENERATE

action_library_version: L1.0

course_time_split: 15/50/15/10

You are an “Individualized Fitness PE Class Plan Generator (Structured Prompt Version)”.

Goal: In a university fitness class (once per week, 90 minutes, 6 weeks, 30-36 students per class), generate an individualized fitness teaching plan for the “first 3 sessions (Weeks 1–3)” for “one single student”, and it must be fully achievable “within class time only” (no after-class exercise assignments allowed).

[Hard rules for reproducibility/auditability (must be followed)]

1) The output must be “deterministic”: the same input must produce the same structure and the same key parameters (exercise names / sets-reps / work-rest / RPE / minute allocation). Do not provide alternative plans (except regressions/substitutions).

2) Exercises can only be selected from the “Exercise Library (L1.0)”; do not invent exercise names or equipment.

3) Minute allocation is fixed and unchanged: warm-up 15' main training 50' accessory 15' cooldown 10' (same for every session).

4) Number rounding rules: minutes = integers; work/rest seconds = integers; sets/rounds/reps = integers; do not output weight/load (unless provided in the input).

5) If there is conflicting information (e.g., two bodyweight values with no dates), you must first output a “Conflict List + Follow-up Questions” and stop generating the plan.

[Input data] See the two tables below (Student_Profile + Performance_Tests). Do not add any free-text information beyond the tables.

|  |  | item | Student A |
| --- | --- | --- | --- |
| Dimension 1: Demographics & Lifestyle | 1 | Age | 20 |
|  | 2 | Gender | Female |
|  | 3 | Sleep quality | Good |
|  | 4 | Pre-exercise food intake | Yes |
|  | 5 | Stress score | 3 |
|  | 6 | Height | 162 |
|  | 7 | Body weight | 48.6 |
| Dimension 2: Physical Activity & Sedentary (IPAQ module) | 8 | Vigorous activity frequency | 1 |
|  | 9 | Vigorous activity duration | 0.75 |
|  | 10 | Moderate activity frequency | 2 |
|  | 11 | Moderate activity duration | 1.75 |
|  | 12 | Walking frequency | 5 |
|  | 13 | Walking duration | 0.75 |
|  | 14 | Average daily steps | 9500 |
|  | 15 | Sedentary time | 6.5 |
| Dimension 3: Preference & Training Background | 16 | Intensity preference | Moderate |
|  | 17 | Exercise barriers | Lack of time, lack of exercise venues or equipment, lack of companions or professional guidance |
|  | 18 | Exercise goal | Weight loss / body shaping |
|  | 19 | Training years | 0.1 |
|  | 20 | Training frequency in the past month | 1 |
|  | 21 | Single-session duration in the past month | 60 |
|  | 22 | Has mastered the big three lifting techniques | Yes |
|  | 23 | Movement proficiency | Proficient |
|  | 24 | Intended training intensity for core movements | RPE 5 |
| Dimension 4: Health & Safety | 25 | Blood pressure | 142/82 |
|  | 26 | Resting heart rate | 95 |
|  | 27 | Fasting blood glucose (FBG) | None |
|  | 28 | Whether liver/kidney dysfunction is present | None |
|  | 29 | Pain / injury history | None |
|  | 30 | PAR-Q red flags | None |
|  | 31 | Chronic disease / medication / allergy | None |
|  | 32 | Doctor-imposed exercise restriction | None |
| Dimension 5: Fitness & Functional Performance (teacher/system imported) | 33 | Total physical test score | 75.1 |
|  | 34 | Vital capacity | 2992 |
|  | 35 | 50 m sprint | 8.8 |
|  | 36 | Standing long jump | 162 |
|  | 37 | Sit-and-reach | 17.9 |
|  | 38 | 800/1000 m | 4.23 |
|  | 39 | Pull-ups / sit-ups | 30 |
|  | 40 | Back strength | 64 |
|  | 41 | Grip strength left | 18.8 |
|  | 42 | Grip strength right | 20.7 |
|  | 43 | Single-leg stance with eyes closed | 180 |

[Unified intensity rules] (must be followed)

- Intensity is expressed with RPE 0–10: 3 = easy, can chat; 5 = breathing faster but can speak in short sentences; 7 = hard, only short words possible; 9 = near maximal.

- In-class goal: prioritize “safety, achievability, and gradual progression”; do not provide medical diagnoses; if red flags appear or pain ≥ 7/10, recommend stopping and seeking professional evaluation.

- No after-class training assignments are allowed; only “lifestyle advice” may be given.

[Exercise Library L1.0 (only the following exercises may be used; if equally suitable, choose the first one in order)]

A Warm-up / Activation: March in place / easy jog, Jumping jacks, High knees, Shoulder circles, Hip circles, World’s greatest stretch, Cat-cow, Glute bridge activation, Deep squat bottom hold 10 sec

B Lower-body main: Bodyweight squat, Box-assisted squat, Single-leg squat, Reverse lunge to knee drive, Step-up, Glute bridge, Single-leg glute bridge, Barbell squat, Hack squat, Romanian deadlift, Single-leg deadlift, Bulgarian split squat, Leg press, Jump squat, Side-lying leg raise, Wall sit

C Upper-body push: Knee push-up, Standard push-up, Incline push-up, Scapular push-up, Bench dip

D Upper-body pull: Bent-over “towel row” isometric fixed object, Resistance band row, Prone Y-T-W, Superman, Reverse fly

E Core anti-extension / stability: Dead bug, Bird dog, Plank, Side plank, Supine crunch, Standing anti-rotation

F Aerobic / Metabolic: Brisk walk / easy run, March in place, Burpee, Jump rope

G Cool-down / Mobility: Calf / quadriceps / glute stretch, Thoracic rotation, Hip flexor stretch, Breathing relaxation.

[Fixed main training structure template (used for every session; parameters individualized)]

- Main training 50': use “3 circuit modules (Block 1 / 2 / 3)”

Each Block contains 4 stations (lower body / upper push / upper pull substitute / core or aerobic)

Default work/rest = 40s/20s; each Block for 2 rounds; 2' rest between Blocks

- Accessory 15': technique cues + low-intensity weak-point work (e.g., core stability / hip-ankle mobility)

- If intensity is too low or the student’s base level is relatively good: 40/20 may be changed to 45/15 or rounds increased to 3 (must follow the “only progression rule”)

[Only progression rule]

- From Session 1 → 2 → 3, only one of the following progression methods is allowed (apply by priority, and only if conditions are met):

1) If no pain and completion rate ≥ 80% and session-RPE ≤ 6: add +1 round to each Block (maximum 3 rounds); work/rest unchanged

2) If completion rate ≥ 80% but RPE < 4: change work/rest to 45/15 (rounds unchanged)

3) If completion rate < 80% or RPE > 7 or pain ≥ 5: reduce rounds by 1 (minimum 2 rounds) or change to 35/25 (choose one only: prioritize reducing rounds)

- Exercise progression is only allowed within the “regression / standard / progression” levels of the same exercise (e.g., knee push-up → standard push-up); do not change to a new exercise unless pain requires substitution.

[Output requirements: must contain two parts]

Part A: YAML (strictly follow the field skeleton; no fields may be added or removed)

Part B: Human-readable version (brief explanation + teaching points)

[YAML field skeleton (must copy the structure exactly)]

meta:

student_id:

prompt_version: "P1.0-GENERATE"

action_library_version: "L1.0"

date_generated:

notes:

profile_summary:

goals:

constraints:

risk_flags:

baseline_highlights:

session_1:

time_split_min: {warmup: 15, main: 50, accessory: 15, cooldown: 10}

rpe_target: ""

warmup: [{name:"", minutes:0, cue:""}]

main_blocks:

- block: 1

work_rest_sec: "40/20"

rounds: 2

stations: [{name:"", cue:"", regression:"", progression:""}]

- block: 2

work_rest_sec: "40/20"

rounds: 2

stations: [{name:"", cue:"", regression:"", progression:""}]

- block: 3

work_rest_sec: "40/20"

rounds: 2

stations: [{name:"", cue:"", regression:"", progression:""}]

accessory: [{name:"", minutes:0, cue:""}]

cooldown: [{name:"", minutes:0, cue:""}]

record_after_class:

all_students: {session_rpe:"", completion_rate:"", effective_main_minutes:""}

hr_substudy_if_worn: {mean_hr:"NA", max_hr:"NA", tiz_minutes:"NA"}

teacher_notes: ""

session_2: {output using the same structure as session_1}

session_3: {output using the same structure as session_1}

adjustment_if_then:

- if: ""

then: ""

mid_replan_data_collection_template:

all_students_fields: ["session_rpe","completion_rate","effective_main_minutes","pain_0_10","notes"]

hr_substudy_fields: ["mean_hr","max_hr","tiz_minutes"]

[Self-checklist (must be included at the end of the output, item by item)]

- All required fields complete and no contradictions: Yes/No (if No, list missing/conflicting items and stop)

- Minute allocation for every session = 15/50/15/10: Yes/No

- All exercises are from Exercise Library L1.0: Yes/No

- Main training contains 3 Blocks, each Block has 4 stations: Yes/No

- Every station includes cue/regression/progression: Yes/No

- No after-class exercise homework assigned: Yes/No

[Consistency protocol (must be executed)]

Generate twice consecutively for the same input; if the two outputs differ in key fields (exercise names / rounds / 40-20 etc. / minute allocation / RPE target):

- Use the version with “exercise appearing earlier in the exercise library order + more conservative parameters (fewer rounds / longer rest)” as the final version;

- Mark in meta.notes: CONSENSUS_APPLIED: Yes.

Start now: first check required fields and contradictions; if incomplete/conflicting → output follow-up question list and stop; if complete → output Part A YAML + Part B brief explanation + self-checklist as required. Translate the Chinese parts into English, keep the rest unchanged.

**Supplementary Appendix 2. Fitness Class Training Plan for 30 College Students.**

meta:

student_id: "CLASS_30_NONSPORTS_COLLEGE"

prompt_version: "P1.0-GENERATE"

action_library_version: "L1.0"

date_generated: "2025-09-14"

notes: "CONSENSUS_APPLIED: No; unified class plan for 30 students, generated as a conservative version for non-sports majors, around 20 years old, with general fitness foundation, taught in a whole-class format."

profile_summary:

goals:

- "Establish foundational movement patterns for the fitness class"

- "Improve class participation and completion rate"

- "Develop basic lower-body strength, upper-body pushing and pulling ability, core stability, and general fitness"

- "Establish a unified rhythm for progression in the following 6-week course"

constraints:

- "30 students taught as one whole class, without individualized grouping"

- "Completed only within class time, with no after-class training assignments"

- "Non-sports major students, with differences in training experience and movement quality"

- "Under large-class organization, prioritize movements that are easy to teach, easy to observe, and easy to rotate"

risk_flags:

- "Default assumption: no PAR-Q red flags; if chest tightness, dizziness, abnormal palpitations, or pain ≥7/10 occurs during class, stop immediately and seek professional evaluation"

- "The first 3 sessions prioritize moderate intensity and movement quality, without pursuing high load"

- "If students have low completion rate or excessively high perceived fatigue, regress according to the unified adjustment rules"

baseline_highlights:

- "Target group is non-sports major college students around 20 years old, with expected large individual differences in fitness foundation"

- "Whole-class teaching prioritizes bodyweight, self-load, and low-complexity organizational movements"

- "The focus of the initial phase is not weight progression, but movement learning, rhythm establishment, and safe completion"

session_1:

time_split_min: {warmup: 15, main: 50, accessory: 15, cooldown: 10}

rpe_target: "5"

warmup:

- {name: "March in place / easy jog", minutes: 4, cue: "Whole class warms up together, gradually entering exercise state, able to talk normally"}

- {name: "Shoulder circles", minutes: 2, cue: "Relax the shoulders, make circles from small to large, prepare for upper-body movements"}

- {name: "Hip circles", minutes: 2, cue: "Keep the pelvis stable, actively mobilize the hips, do not twist the waist"}

- {name: "World’s greatest stretch", minutes: 3, cue: "Front foot stable, rear leg extended, thoracic spine opens up"}

- {name: "Cat-cow", minutes: 2, cue: "Move the spine segment by segment, slowly, coordinated with breathing"}

- {name: "Glute bridge activation", minutes: 2, cue: "Drive through the heels, brace the core, squeeze the glutes for 1 second at the top"}

main_blocks:

- block: 1

work_rest_sec: "40/20"

rounds: 2

stations:

- {name: "Bodyweight squat", cue: "Keep heels grounded, knees aligned with toes, exhale as you stand up", regression: "Box-assisted squat", progression: "Barbell squat"}

- {name: "Incline push-up", cue: "Keep the body in a straight line, control the lowering phase, avoid shrugging when pushing up", regression: "Knee push-up", progression: "Standard push-up"}

- {name: "Prone Y-T-W", cue: "Keep the neck neutral, retract and depress the shoulder blades, make the movement small and precise", regression: "Superman", progression: "Reverse fly"}

- {name: "Dead bug", cue: "Keep lower back on the floor, slowly extend opposite arm and leg, keep the abdomen braced", regression: "Supine crunch", progression: "Plank"}

- block: 2

work_rest_sec: "40/20"

rounds: 2

stations:

- {name: "Reverse lunge to knee drive", cue: "Step back softly, drive through the front foot to stand, keep balance on return", regression: "Step-up", progression: "Bulgarian split squat"}

- {name: "Scapular push-up", cue: "Keep arms straight, only protract and retract the shoulder blades, avoid sagging at the waist", regression: "Incline push-up", progression: "Standard push-up"}

- {name: "Superman", cue: "Reach the limbs away, engage the back, keep a moderate lift off the ground", regression: "Prone Y-T-W", progression: "Reverse fly"}

- {name: "March in place", cue: "Keep a steady cadence, swing the arms naturally, breathing should be faster but short sentences are still possible", regression: "March in place / easy jog", progression: "Jump rope"}

- block: 3

work_rest_sec: "40/20"

rounds: 2

stations:

- {name: "Glute bridge", cue: "Drive through the heels to lift the hips, keep the pelvis neutral, squeeze the glutes at the top", regression: "Side-lying leg raise", progression: "Single-leg glute bridge"}

- {name: "Knee push-up", cue: "Keep the torso in a straight line, elbows about 45 degrees, brace the core when pushing up", regression: "Incline push-up", progression: "Standard push-up"}

- {name: "Reverse fly", cue: "Hinge at the hips, open both arms to the sides until shoulder level", regression: "Prone Y-T-W", progression: "Resistance band row"}

- {name: "Plank", cue: "Head, shoulders, hips, and ankles in one line, brace the abdomen and glutes, keep natural breathing", regression: "Dead bug", progression: "Side plank"}

accessory:

- {name: "Deep squat bottom hold 10 sec", minutes: 5, cue: "Practice bottom squat control together, keep heels down, lift the chest"}

- {name: "Bird dog", minutes: 5, cue: "Extend opposite limbs, keep the pelvis stable, reinforce class-wide core control"}

- {name: "Hip circles", minutes: 5, cue: "Low-intensity hip mobility work to support recovery"}

cooldown:

- {name: "Calf / quadriceps / glute stretch", minutes: 4, cue: "Stretch each area slowly, no bouncing"}

- {name: "Hip flexor stretch", minutes: 2, cue: "Slight posterior pelvic tilt, feel the stretch in the front side"}

- {name: "Thoracic rotation", minutes: 2, cue: "Actively rotate the upper back, relax shoulders and neck"}

- {name: "Breathing relaxation", minutes: 2, cue: "Inhale for 4 seconds, exhale for 6 seconds, gradually stabilize heart rate"}

record_after_class:

all_students: {session_rpe: "0-10", completion_rate: "0-100%", effective_main_minutes: "min"}

hr_substudy_if_worn: {mean_hr: "NA", max_hr: "NA", tiz_minutes: "NA"}

teacher_notes: "In the first session, focus on observing whole-class movement understanding, rotation order, completion rate, and RPE distribution, to provide evidence for adjustments in session 2."

session_2:

time_split_min: {warmup: 15, main: 50, accessory: 15, cooldown: 10}

rpe_target: "5-6"

warmup:

- {name: "March in place / easy jog", minutes: 4, cue: "Enter exercise state more smoothly than in the first session"}

- {name: "Shoulder circles", minutes: 2, cue: "Prepare for pushing and pulling movements, avoid shrugging"}

- {name: "Hip circles", minutes: 2, cue: "Prepare for multi-joint lower-body movements, control the pelvis"}

- {name: "World’s greatest stretch", minutes: 3, cue: "Increase hip and thoracic mobility, maintain fluent movement"}

- {name: "Cat-cow", minutes: 2, cue: "Spinal movement coordinated with breathing, keep a stable rhythm"}

- {name: "Glute bridge activation", minutes: 2, cue: "Continue establishing glute-first activation sequence"}

main_blocks:

- block: 1

work_rest_sec: "40/20"

rounds: 2

stations:

- {name: "Bodyweight squat", cue: "Compared with the first session, further emphasize squat depth and stability", regression: "Box-assisted squat", progression: "Barbell squat"}

- {name: "Incline push-up", cue: "Control the lowering speed, keep the body in one line when pushing up", regression: "Knee push-up", progression: "Standard push-up"}

- {name: "Prone Y-T-W", cue: "Complete each letter clearly, stabilize the shoulder blades first", regression: "Superman", progression: "Reverse fly"}

- {name: "Dead bug", cue: "Slow down the movement, extend the limbs farther without letting the lower back sag", regression: "Supine crunch", progression: "Plank"}

- block: 2

work_rest_sec: "40/20"

rounds: 2

stations:

- {name: "Reverse lunge to knee drive", cue: "Step back more steadily, return to standing with better single-leg control", regression: "Step-up", progression: "Bulgarian split squat"}

- {name: "Scapular push-up", cue: "Emphasize shoulder blade protraction and retraction without elbow bending", regression: "Incline push-up", progression: "Standard push-up"}

- {name: "Superman", cue: "Reach hands and feet away, engage the back evenly, pause for 1 second", regression: "Prone Y-T-W", progression: "Reverse fly"}

- {name: "March in place", cue: "Slightly increase cadence while maintaining moderate breathing load", regression: "March in place / easy jog", progression: "Jump rope"}

- block: 3

work_rest_sec: "40/20"

rounds: 2

stations:

- {name: "Glute bridge", cue: "Hold the top for 2 seconds, reduce lower-back compensation", regression: "Side-lying leg raise", progression: "Single-leg glute bridge"}

- {name: "Knee push-up", cue: "Use full range of motion, keep the core stable, avoid sagging at the waist", regression: "Incline push-up", progression: "Standard push-up"}

- {name: "Reverse fly", cue: "Open both arms to shoulder level, keep the movement path stable", regression: "Prone Y-T-W", progression: "Resistance band row"}

- {name: "Plank", cue: "Maintain one straight line, breathe normally, avoid shrugging", regression: "Dead bug", progression: "Side plank"}

accessory:

- {name: "Deep squat bottom hold 10 sec", minutes: 5, cue: "Reinforce class-wide bottom squat control and breathing rhythm"}

- {name: "Bird dog", minutes: 5, cue: "Improve trunk stability and limb coordination"}

- {name: "Cat-cow", minutes: 5, cue: "Low-intensity spinal mobility recovery as technique consolidation"}

cooldown:

- {name: "Calf / quadriceps / glute stretch", minutes: 4, cue: "Complete cool-down stretching under unified class instruction"}

- {name: "Hip flexor stretch", minutes: 2, cue: "Keep the torso upright and open the front hip"}

- {name: "Thoracic rotation", minutes: 2, cue: "Use gentle movement and natural breathing"}

- {name: "Breathing relaxation", minutes: 2, cue: "Lengthen exhalation to promote recovery"}

record_after_class:

all_students: {session_rpe: "0-10", completion_rate: "0-100%", effective_main_minutes: "min"}

hr_substudy_if_worn: {mean_hr: "NA", max_hr: "NA", tiz_minutes: "NA"}

teacher_notes: "If most students achieved completion rate ≥80% and session-RPE ≤6 in the first session, unified progression may be considered in session 3."

session_3:

time_split_min: {warmup: 15, main: 50, accessory: 15, cooldown: 10}

rpe_target: "6"

warmup:

- {name: "March in place / easy jog", minutes: 4, cue: "Enter moderate-intensity state smoothly"}

- {name: "Shoulder circles", minutes: 2, cue: "Relax the upper body and prepare for pushing and pulling movements"}

- {name: "Hip circles", minutes: 2, cue: "Actively mobilize the hips while maintaining pelvic control"}

- {name: "World’s greatest stretch", minutes: 3, cue: "Increase hip and thoracic coordination, keep movement fluent"}

- {name: "Cat-cow", minutes: 2, cue: "Activate spinal control with even movement"}

- {name: "Glute bridge activation", minutes: 2, cue: "Maintain glute-first activation and reduce lower-back compensation"}

main_blocks:

- block: 1

work_rest_sec: "40/20"

rounds: 2

stations:

- {name: "Bodyweight squat", cue: "Maintain consistent depth and rhythm, focus on knee-hip coordination", regression: "Box-assisted squat", progression: "Barbell squat"}

- {name: "Incline push-up", cue: "Push up fully, stabilize the shoulders, keep the body in a straight line", regression: "Knee push-up", progression: "Standard push-up"}

- {name: "Prone Y-T-W", cue: "Do not use momentum, make shoulder blade control clearer", regression: "Superman", progression: "Reverse fly"}

- {name: "Dead bug", cue: "Continue slowing down the movement to improve core control quality", regression: "Supine crunch", progression: "Plank"}

- block: 2

work_rest_sec: "40/20"

rounds: 2

stations:

- {name: "Reverse lunge to knee drive", cue: "Emphasize stable return position and single-leg balance", regression: "Step-up", progression: "Bulgarian split squat"}

- {name: "Scapular push-up", cue: "Actively glide the shoulder blades while keeping the ribs tucked", regression: "Incline push-up", progression: "Standard push-up"}

- {name: "Superman", cue: "Lift to a consistent height and engage the back evenly", regression: "Prone Y-T-W", progression: "Reverse fly"}

- {name: "March in place", cue: "Maintain aerobic intensity close to RPE 6, but do not overshoot", regression: "March in place / easy jog", progression: "Jump rope"}

- block: 3

work_rest_sec: "40/20"

rounds: 2

stations:

- {name: "Glute bridge", cue: "Keep the pelvis stable and make top-position force production clearer", regression: "Side-lying leg raise", progression: "Single-leg glute bridge"}

- {name: "Knee push-up", cue: "Stay stable throughout and perform with standard form to prepare for later progression", regression: "Incline push-up", progression: "Standard push-up"}

- {name: "Reverse fly", cue: "Stabilize the shoulder blades first, then open the arms, keep the movement path clear", regression: "Prone Y-T-W", progression: "Resistance band row"}

- {name: "Plank", cue: "Keep head, shoulders, hips, and ankles in one line, avoid holding the breath", regression: "Dead bug", progression: "Side plank"}

accessory:

- {name: "Deep squat bottom hold 10 sec", minutes: 5, cue: "Continue consolidating squat posture and bottom-position control"}

- {name: "Bird dog", minutes: 5, cue: "Reinforce core stability and four-limb coordination"}

- {name: "Glute bridge activation", minutes: 5, cue: "Low-intensity additional glute activation to support movement consolidation"}

cooldown:

- {name: "Calf / quadriceps / glute stretch", minutes: 4, cue: "Perform lower-body relaxation together with slow movement"}

- {name: "Hip flexor stretch", minutes: 2, cue: "Stabilize the pelvis and relax the front hip"}

- {name: "Thoracic rotation", minutes: 2, cue: "Release upper-back tension and keep relaxed breathing"}

- {name: "Breathing relaxation", minutes: 2, cue: "Gradually lower heart rate and end the class"}

record_after_class:

all_students: {session_rpe: "0-10", completion_rate: "0-100%", effective_main_minutes: "min"}

hr_substudy_if_worn: {mean_hr: "NA", max_hr: "NA", tiz_minutes: "NA"}

teacher_notes: "After session 3, decide whether the later class should uniformly progress from 2 rounds to 3 rounds, or continue maintaining the current version, based on overall class completion rate and RPE."

adjustment_if_then:

- if: "Most students in the class have no pain and completion_rate≥80% and session_rpe≤6"

then: "In the next class, add +1 round to each Block, up to a maximum of 3 rounds, while keeping work_rest_sec at 40/20 unchanged"

- if: "Most students in the class have completion_rate≥80% and session_rpe<4"

then: "In the next class, adjust work_rest_sec to 45/15, with rounds unchanged"

- if: "A relatively large number of students in the class have completion_rate<80% or session_rpe>7 or pain_0_10≥5"

then: "In the next class, first prioritize reducing each Block by 1 round but not below 2 rounds; if still not suitable, then change to 35/25"

mid_replan_data_collection_template:

all_students_fields: ["session_rpe","completion_rate","effective_main_minutes","pain_0_10","notes"]

hr_substudy_fields: ["mean_hr","max_hr","tiz_minutes"]
